# COVID-19: Protecting Healthcare Workers is a priority

**DOI:** 10.1017/ice.2020.148

**Published:** 2020-04-17

**Authors:** Francesco Chirico, Gabriella Nucera, Nicola Magnavita

**Affiliations:** 1 School of Occupational Medicine, Università Cattolica del Sacro Cuore, Rome, Italy; 2 Health Service Department, State Police, Ministry of Interior, Milan, Italy; 3 University of Milan, Milan, Italy; 4 ASST Fatebenefratelli Sacco, FatebeneFratelli Hospital, Milan, Italy; 5 Department of Woman/Child and Public Health, Fondazione Policlinico “A.Gemelli” IRCCS, Rome, Italy

*To the Editor*—We very much appreciated the letter by Zhou et al^1^ regarding the protection of Chinese healthcare workers (HCWs) while fighting the COVID-19 pandemic. The authors recognized that the lack of awareness and training, the shortage of personal protective equipment (PPE), and the lack of point-of-care diagnostic tests for were the most important sources of viral spread. In Italy, more infections among HCWs have been recorded than in China. As of April 5, 2020, 12,252 HCWs in Italy had tested positive for SARS-CoV-2, comprising 10% of Italy’s COVID-19 cases^2^; furthermore, 80 medical doctors and 25 nurses had died. Notably, official figures probably underestimate the real impact of COVID-19 on Italian HCWs because many have not been tested and a large majority of coronavirus infections do not result in symptoms or remain paucisymptomatic.^3^ In Italy, HCWs are facing the same issues that Zhou highlighted in Chinese hospitals. SARS-CoV-2 has a high transmissibility rate in indoor environments and, therefore, asymptomatic patients admitted to hospitals without respiratory symptoms have probably spread the virus to unaware and unprotected HCWs. These HCWs have, in turn, infected other patients, visitors, and staff, further amplifying viral transmission. It is well-known that a hospital may amplify an epidemic and that epidemics may overwhelm a hospital’s capacity to deliver healthcare services.^4^ Therefore, in addition to general lockdown and social distancing measures, protecting HCWs is a priority in alleviating the burden on the hospitals. However, in the absence of effective therapies or a vaccination, before the onset of further COVID-19 waves, it is important to relocate the public health emergency response from the hospitals to other locations by integrating the hospital into an overall epidemic response.^4^ In this regard, communication and mass-media information campaigns for the public are crucial.
